# Supplementary material for: Salt-inducible kinases (SIKs) regulate TGFβ-mediated transcriptional and apoptotic responses
Source: Cell Death Dis. 2020 Jan 22;11(1):49. doi: 10.1038/s41419-020-2241-6 (PMC6976658; doi:10.1038/s41419-020-2241-6)
Supplement: Supplementary file 1 — Supplementary Figure Legends [file 41419_2020_2241_MOESM1_ESM.docx]

**Salt-inducible kinases (SIKs) regulate TGFβ-mediated transcriptional and apoptotic responses**

Luke D. Hutchinson^1^, Nicola J. Darling^1^, Stephanos Nicolaou^2,#^, Ilaria Gori^2^, Daniel R. Squair^1^, Philip Cohen^1^, Caroline S. Hill^2^ and Gopal P. Sapkota^1^*

**Supplementary Figures and Legends:**

**Figure S1. *In vitro* phosphorylation of recombinant SMAD proteins**

**A:** *In vitro* protein kinase assay analysis using the recombinant kinases MBP-SIK1, GST-SIK2 and GST-SIK3 and either recombinant SMAD2, SMAD3 or SMAD4 as the substrate. A constitutively active mutant form of the type I TGFβ receptor (GST-TGFβRI^T204D^) was included as a positive control.

**Figure S2.**

**A:** *In vitro* protein kinase assay analysis using the recombinant kinases GST-TGFβRI^T204D^, GST-SIK2 and GST-SIK3 and cleaved recombinant SMAD3 as the substrate. The small-molecule inhibitors SB-505124 and HG-9-91-01 were included as controls.

**B:** Immunoblot analysis of wild type HaCaT cells and SMAD3^-/-^ cells following incubation with recombinant human TGFβ_1_. Cell lysates were resolved via SDS-PAGE and membranes subjected to immunoblotting with the indicated antibodies.
