# Supplementary figures and images for: Salt-inducible kinases (SIKs) regulate TGFβ-mediated transcriptional and apoptotic responses
Source: Cell Death Dis. 2020 Jan 22;11(1):49. doi: 10.1038/s41419-020-2241-6 (PMC6976658; doi:10.1038/s41419-020-2241-6)

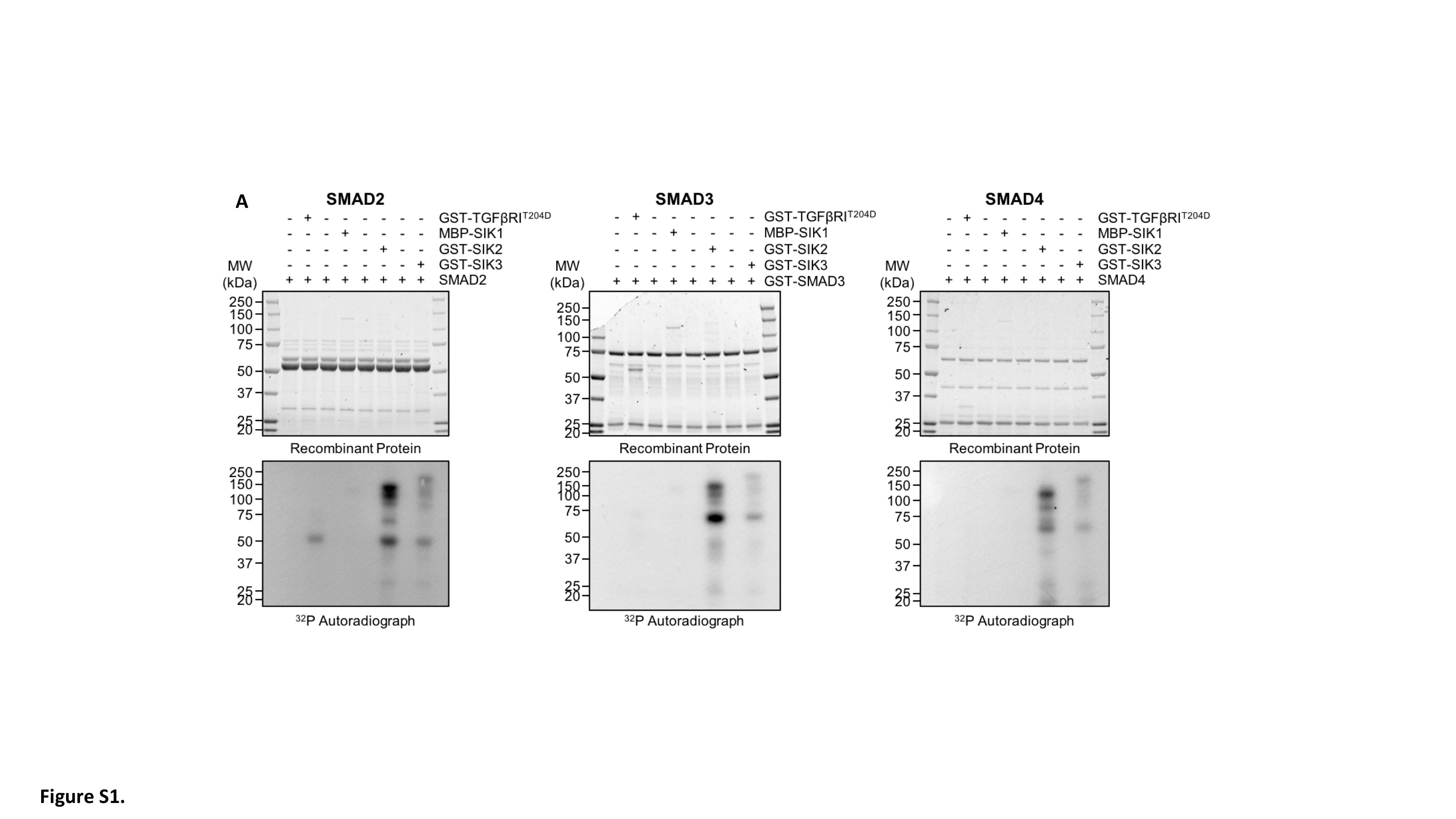

Supplement: Supplementary file 2 — Figure S1 [file 41419_2020_2241_MOESM2_ESM.tif]

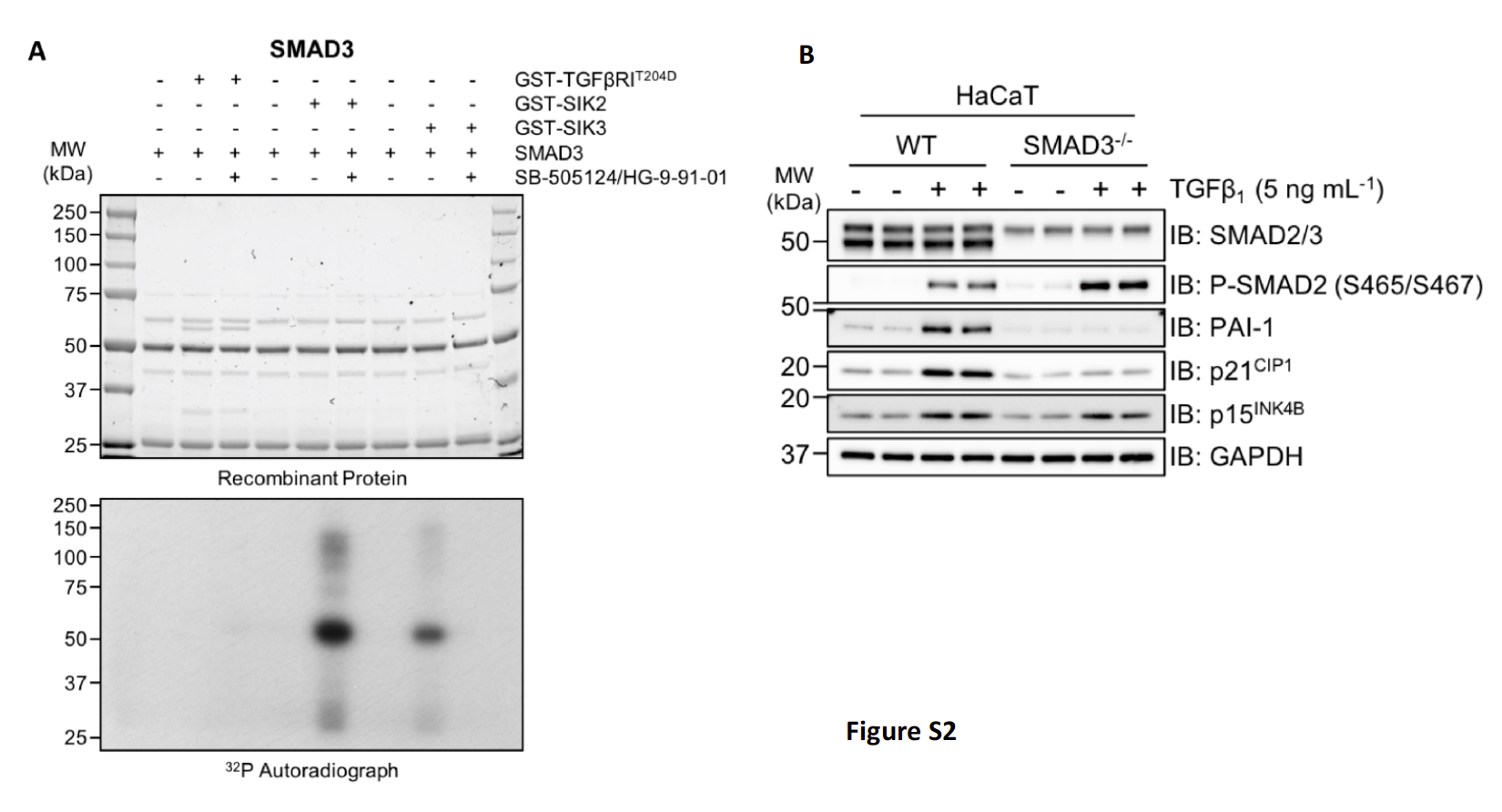

Supplement: Supplementary file 3 — Figure S2 [file 41419_2020_2241_MOESM3_ESM.tif]
